# South Vietnamese Rural Mothers' Knowledge, Attitude, and Practice in Child Health Care

**DOI:** 10.1155/2016/9302428

**Published:** 2016-01-03

**Authors:** Dinh Thac, Freddy Karup Pedersen, Tang Chi Thuong, Le Bich Lien, Nguyen Thi Ngoc Anh, Nguyen Ngoc Phuc

**Affiliations:** ^1^Children's Hospital 1 (CH1), 341 Su Van Street, District 10, Ho Chi Minh City, Vietnam; ^2^The Juliane Marie Centre, University Clinic of Children and Adolescents, Rigshospitalet, Blegdamsvej 9, 2100 Copenhagen, Denmark; ^3^Ho Chi Minh Child Welfare Association, 85/65 Pham Viet Chanh Street, Binh Thanh District, Ho Chi Minh City, Vietnam

## Abstract

A study of 600 rural under-five mothers' knowledge, attitude, and practice (KAP) in child care was performed in 4 southern provinces of Vietnam. The mothers were randomly selected and interviewed about sociodemographic factors, health seeking behaviour, and practice of home care of children and neonates. 93.2% of the mothers were literate and well-educated, which has been shown to be important for child health care. 98.5% were married suggesting a stable family, which is also of importance for child health. Only 17.3% had more than 2 children in their family. The mother was the main caretaker in 77.7% of the families. Only 1% would use quacks as their first health contact, but 25.2% would use a private clinic, which therefore eases the burden on the government system. Nearly 69% had given birth in a hospital, 27% in a commune health station, and only 2.7% at home without qualified assistance. 89% were giving exclusive breast feeding at 6 months, much more frequent than in the cities. The majority of the mothers could follow IMCI guideline for home care, although 25.2% did not deal correctly with cough and 38.7% did not deal correctly with diarrhoea. Standard information about Integrated Management of Childhood Illnesses (IMCI) based home care is still needed.

## 1. Introduction

To improve infant and child morbidity and mortality in Vietnam, information about mothers' KAP in relation to child health care in the community at commune and village level is needed. Mothers' KAP can provide a reliable picture of child care in communities like health seeking behaviour of mothers, practices of home care, and recognition of danger signs of childhood illness. In addition, mothers' KAP of delivery and neonatal care are important [1]. A study at Department of Paediatrics of a tertiary care hospital in South India indicated that awareness and attitude of postnatal mothers towards neonatal care have lots of lacunae especially in those who belong to the lower socioeconomic groups [2]. Furthermore, newborn deaths are reported to account for up to 40% of all deaths in children under 5. According to UNICEP's child health statistics in Vietnam, the under-five mortality rate (U5MR) has decreased from 51/1000 live births in 1990 to 23/1000 in 2012, while infant mortality rate (under 1) is 36/1000 in 1990 and this rate has decreased a half with 18/1000 in 2012. In particular, neonatal mortality rate accounts for 12/1000 in 2012 that is still quite high in developing countries. The significant decrease in under-five mortality among other factors has been associated with implementation of the Integrated Management of Childhood Illness (IMCI) program throughout the country with training of health staff, upgrading of health facilities, and health education of under-five mothers to deal with the 5 most frequent causes of deaths in Vietnamese children. The results of the IMCI program in terms of the mothers knowledge, attitude, and practice (KAP), however, have not been systematically studied and the program does not include any follow-up or refresher training, which is likely to be of importance for the sustainability of the KAP obtained through the program. The present study aims at providing information about the status of KAP of mothers regarding “the IMCI program of the 5 common diseases” approximately 6 months after they have participated in the training course on the IMCI guidelines home care. The findings of a KAP study can be considered as indicators for strategic interventions to improve child health and reduce morbidity and mortality including neonatal mortality. This information will be important for the authorities decisions on the need for additional health education of mothers and for which subjects to prioritize in such education. There is scope for improvement by providing better care and health education for antenatal mothers and formal education has a positive influence on the KAP of the mothers [2, 3]. The objective of this study was to provide trustworthy information related to mothers' KAP about child care at home and in community, which can be used to set up appropriate interventions for improving child health in Vietnam [4].

## 2. Materials and Methods

*The overall objective of this study was *to provide trustworthy information related to mothers' KAP about child health care at home and in community that can be used to set up appropriate interventions for improving child health in Vietnam.

*The specific objectives were as follows:*

1. to study KAP of mothers and caretakers in dealing with fever, diarrhoea, and cough in their children and in their health seeking behaviour;
2. to investigate KAP of mothers and caretakers regarding newborn babies' care, including breast feeding, eye care, skin care, umbilicus care, jaundice, prevention of hypothermia, and neonatal tetanus;
3. to suggest appropriate interventions to improve child health.

### 2.1. Study Design

A cross-sectional descriptive study.

### 2.2. Methods and Material

The data collection for the study has taken place as structured interviews with 600 rural mothers in the 4 southern provinces of Dong Thap, Vinh Long, Lam Dong, and Ho Chi Minh City of Vietnam on average of 6 months (5–7 months) after IMCI health education had taken place. In each of the 4 project provinces, 150 KAP study questionnaires were completed, that is, 15 questionnaires per health commune station from 10 health commune stations in each of the 4 provinces, that were randomly selected among all health communes of the province.

Mothers or guardians who had children under 5 years of age living in the project areas and who were judged as being able to answer a questionnaire were included. There was no limitation of age, educational level, or socioeconomic status.

Mothers participating in the study were selected by using a name list of all mothers managed by the commune health station for the national child health programs or the Expanded Program of Immunization (EPI); a table of random numbers and mother's name list with an ID number given by the health staff were used for the final selection until the number required in the sample was reached.

No mothers or caretakers were judged or proved as being unable to answer the questionnaire, but 9 of the mothers selected from the randomization list could not be met, because they were living with family or working in another province at the period of the interviews. They were substituted by 9 other mothers from the randomization list.

Answers were registered as correct if completely in accordance with IMCI guidelines. In case the mother after the IMCI health education had handled her child when sick, this also had to have been done in accordance with the guidelines for the conditions studied for the mother to be registered as correct. No direct observations of the mother's actions were performed.

All data collected were computerized anonymously in Access in Vietnamese by the Project Office of Children's Hospital in Ho Chi Minh City (CH1). 10% of questionnaires were entered twice; a maximum 5% discrepancy was accepted. The chief doctor of the commune health station checked the quality of the data before sending them to the KAP study's group of CH1 for entering data anonymously. Data entry and analysis for the KAP study were performed by EpiData Entry 3.1 software and by Stata 10 version software.

### 2.3. Ethical Approval

Informed consent was obtained from mother or guardian before enrolment. The Scientific Research Unit and Ethical Committee Board of CH1 and the Danish Data Protection Agency approved the study. Local authorities and Provincial Health Services' Ethical Committee Board gave permission to carry out the study.

## 3. Results

600 mothers and caretakers from 40 communes in 4 southern Vietnamese provinces (10 communes in each province) were selected in a randomized way and interviewed with the following results.

As may be seen from Table 1, 93.2% of the mothers were literate and well-educated, and 98.5% were married. Only 17.3% had more than 2 children in their family.  Table 2 shows that the mother was the main caretaker in 77.7% of the families. Only 1% would use quacks as their first health contact, but 25.2% would use a private clinic; nearly 69% had given birth in a hospital and 27% in a commune health station and only 2.7% at home without qualified assistance.

83.5% of mothers knew the proper way of caring for a sick child with fever by taking an antipyretic like paracetamol to reduce temperature, 61.3% could give children with acute diarrhoea a lot of boiled or clean water (prior to oral rehydration solution, ORS) to improve loss of water, and 74.8% could make safe traditional medicine to improve a child's cough. There were 60.7% of newborn babies receiving early breast feeding (30 minutes right after birth), and there were 87.5% of all mothers understanding how to prevent hypothermia like wearing sweater, woollen stockings, using electrical fan, or controlling air conditioning more properly. Regarding newborn babies with abnormal jaundice, there were 94% of mothers who confirmed that the baby had to be taken to see a doctor as soon as possible. 94.7% of mothers knew how to prevent neonatal tetanus infection by receiving two doses of vaccine against tetanus (VAT) during pregnancy.

The characteristics of the interviewees are given in Table 1.

Child health care practices as reported by the interviewees are given in Table 2.

**Figure 1:**
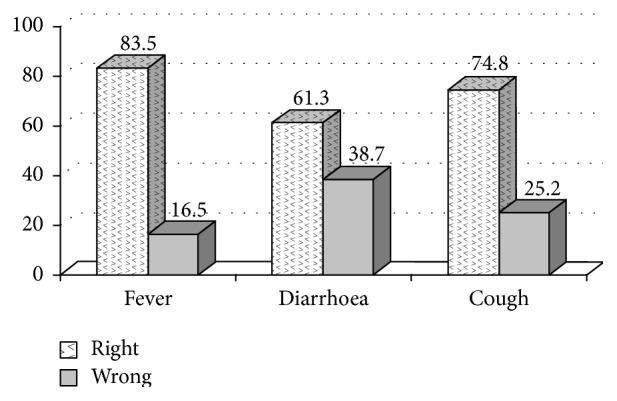
Mother's basic care for sick children.

Figure 1 illustrates the quality of the mothers' home care of fever, diarrhoea, and cough as compared to IMCI guidelines.

**Figure 2:**
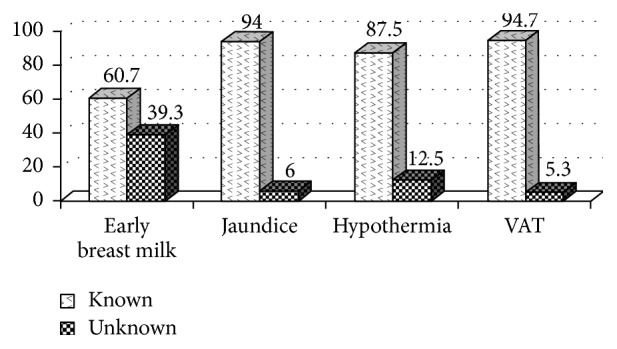
Mother's basic care for newborn babies.

In Figure 2, the qualities of mothers' care for the newborns are illustrated.

**Figure 3:**
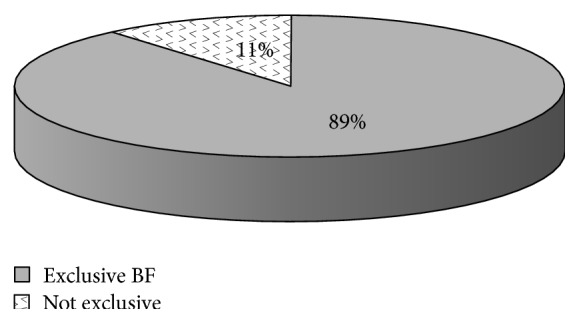
Exclusive breast feeding (BF) at 6 months.

As illustrated in Figure 3, there were nearly 89% of mothers in this KAP study practising exclusive breast feeding during the first 6 months of life.

## 4. Discussion 

This study was performed in 2 Mekong Delta provinces (Dong Thap and Vinh Long), 1 southern highland province (Lam Dong), and two rural districts of Ho Chi Minh City. As 4 different southern provinces were included, because the sample is large and because both commune health stations and mothers selected to participate were chosen randomly, it is likely that the results found are representative, at least for the situation in most provinces of southern Vietnam. Only 9 of the first 600 selected mothers were unable to participate because of absence, but they could be substituted in the same randomized way, which also lends credibility to the representation. The large sample size, the randomized methodology, and the complete lack of nonconsenters add strength to the study.

Weaknesses are that abilities and practices were not directly observed and that practice was only evaluated as it was recalled, only in those who had dealt with a relevant situation within the 6-month period after health education. The relatively short period of 6 months after the health education also limit the usefulness of the findings, as these may be less favourable after a longer period.

The findings of both a very high literacy rate (93.2%) in the caretakers and a very high percentage of married caretakers (98.5%) suggesting a stable family situation have been shown to be related to good child health in other studies [5, 6]. The same is the case for the relatively small family size found [7], with only 17.3% having more than 2 children. The fact that the mother being the caretaker in 77.7% of the cases probably adds to this and is likely to be a reflection of the rural environment, where few women have outside work (75.7% were found to be farmers or housewives). Presumably, it has a similarity to a study in rural Thailand [8]. The culturally determined sociodemographic factors in rural southern Vietnam are thus favouring good child health.

Health care seeking behaviour is of importance for the quality of treatment of sick children [1, 3, 9]. A tradition for seeking advice from local healers or quacks first may cause delayed adequate treatment as may buying of the counter drugs in private pharmacies for self-treatment [1]. The very large majority of caretakers in this study, however, exhibited a relevant and rational care seeking behaviour, turning to commune health station or hospital in three quarters of the cases, with the remaining ones almost all using private general practitioners that in South Vietnam are licensed doctors. The choice of private practitioners may be influenced by proximity and the timesaving effect of the possibility to make appointments. It does help to ease the burden on the public-free services for children below 6 years without affecting quality of treatment, but of course it is available only to those who are able to pay.

The very high percentage of mother's breast feeding at 6 months (89%) and 60.7% of mothers giving their babies early breast feeding (30 minutes right after birth) probably also reflect the fact that most mothers in rural South Vietnam are farmers or housewives (75.7% as mentioned above), with few working outside the home setting and probably also being less exposed to formula advertisements than their urban counterparts. This finding may be expected to positively influence child health in rural Vietnam, in contrast to the general prevalence, because the prevalence of exclusive breast feeding in Vietnam and several Southeast Asian countries is very low now [10–13]. France Begin, UNICEF Nutrition Advisor for East Asia and the Pacific, is quoted as saying “The falling rates of breast feeding across East Asia are alarming. In Thailand as little as 5% of all mothers breastfeed while the rate is less than 20% in Vietnam. In China, only 28% of babies are breastfed.”

With 69% of mothers giving birth in hospital and 27% giving birth in commune health station and only 2.7% giving birth at home, the World Health Organization (WHO) recommendation of delivery in the presence of a skilled birth attendant is very close to be fulfilled in Vietnam. A further reduction of perinatal mortality therefore must rest on training and equipping staff at health institutions and improving cooperation between obstetricians and paediatricians [6].

Any appropriate treatment of a sick child starts with the caretakers' ability to recognise the situation and deal with it correctly. This study found that 25% did not deal correctly with cough and 38% did not deal correctly with diarrhoea. In particular, the latter is surprising in view of the many health education efforts over the years with relation to oral rehydration. Our study, however, shows that there is a need for continued follow-up of IMCI health education on village level [4, 14, 15].

Knowledge about basic neonatal care activities like controlling abnormal jaundice or preventing hypothermia was practiced quite correctly by almost all mothers, including prevention of neonatal tetanus infection by receiving two doses of vaccine against tetanus during the pregnant period. The importance of health education in the practice of vaccination for maternal and child health is an important intervention for improving child health and appeared to have been effective in this study [5, 16].

## 5. Conclusion

This study shows a very high rate of literacy, married status, small family size, rational health seeking behavior, delivery with a skilled birth attendant, and exclusive breast feeding up to 6 months in rural mothers in southern Vietnam, all important for child health.

Knowledge, attitude, and practice of IMCI home care guidelines were good in village caretakers for many, especially neonatal issues, but 25% could not deal correctly with cough and 38% could not deal correctly with diarrhea, both important for under-five morbidity and mortality. There is therefore a need for follow-up of IMCI health education on village level in southern Vietnam.

## Figures and Tables

**Table 1:** Basic characteristics related to all interviewees.

Variable	Number of interviewees *N* = 600	Percentage
Sex		
Female	596	99.3
Male	4	0.7
Age (years)		
Number of mothers, 28 years old (mean age)	54	9.0
Eldest person interviewed (71 years old)		
Youngest one interviewed (17 years old)		
Education level		
Illiteracy	41	6.8
Primary school	207	34.5
Secondary school	214	35.7
High school	123	20.5
College/university	15	2.5
Occupation		
Farmer	304	50.7
Housework	150	25.0
Business person	73	12.2
Worker	26	4.3
Teacher	19	3.2
Health staff	6	1.0
Economic status		
Poor^*∗*^	147	24.5
Average	429	71.5
Rich	17	2.8
Unknown	7	1.2
Marital status		
Single	2	0.3
Married	591	98.5
Divorced	4	0.7
Widowed	3	0.5
Siblings in a family		
01 child	215	35.8
02 children	281	36.8
03 children	84	14.0
04 children	20	3.3

^*∗*^Definition of household's economic status: based on Prime Minister's official paper number 09/2011/QĐ-TTg, dated November 30, 2011, for a standard to define a poor household in Vietnam: a rural household's income  is less than 400,000 VND per month (equivalence is 20 USD) or 4,800,000 VND per year (about 230 USD); an urban household's income is less than 500,000 VND per month (equivalence is 24 USD) or 6,00,000 VND per year (about 286 USD).

**Table 2:** Child health care practices at home.

Variable	Number of interviewees *N* = 600	Percentage
Child care mainly by		
Mother	466	77.7
Father	55	9.2
Grand parents	39	6.5
Others (uncle, aunt)	40	6.6
First place where a sick child was taken in order to get treatment		
Health commune station	346	57.7
Private practice clinic	151	25.2
Provincial/district hospital	97	16.2
Quack doctor	6	1.0
Reason for choosing health commune station as first place to get treatment		
Short distance	173	28.8
Familiar place	205	34.2
Free of charge for under 6 years old	136	22.7
Place for mothers to give birth		
Health commune station	162	27.0
District hospital	193	32.2
General provincial hospital	220	36.7
Private practice clinics	9	1.5
Traditional Birth Attendant (TBA)	16	2.7
Breast feeding right after birth		
30 minutes after birth	364	60.7
1 hour after birth	172	28.7
12 hours after birth	24	4.0
The following day	38	6.3
No breast feeding	2	0.3
Prevention of neonatal tetanus by vaccine against antitetanus (VAT) injection		
Understanding clearly	568	94.7
Unknown	32	5.3
